# Revisiting the question: When is a centromere not a kinetochore?

**DOI:** 10.1007/s10577-025-09782-2

**Published:** 2025-10-25

**Authors:** Samuel Corless, Gokilavani Thangavel, Sylvia Erhardt

**Affiliations:** 1 University of Edinburgh, Institute of Cell Biology, Michael Swann Building, Max Born Crescent, Edinburgh, EH9 3BF UK; 2 Molecular Cell Biology, Karlsruhe Institute of Technology (KIT) - Zoological Institute, Kaiserstrasse 12, 76131 Karlsruhe, Germany; 3 Karlsruhe Institute of Technology (KIT) – Institute of Biological and Chemical Systems-Functional Molecular Systems, Karlsruhe, Germany

**Keywords:** Centromere, Kinetochore, Chromatin organisation, Satellite DNA

## Abstract

Centromeres have been the focus of extensive research for almost a century, so it may come as a surprise that a consistent definition and nomenclature for these structures remains elusive. In recent times, centromeric chromatin is most frequently defined by the presence of nucleosomes containing the H3 variant CENP-A and is typically synonymous with the site of the inner-kinetochore. However, crucial mammalian centromere proteins including CENP-B and INCENP have well defined distributions that show very little overlap with CENP-A. Additional protein localisations spanning the primary constriction or forming a band below CENP-A chromatin have been reported. Together, these observations suggest a complex and multi-layered chromatin organisation that is not well served by the canonical dichotomy of ‘centromeric’ and ‘pericentromeric’ chromatin. Strikingly, this is not a new observation but was made soon after the discovery of CENP proteins, including in a 1991 publication titled ‘When is the centromere not a kinetochore?’. Here we revisit this question, which has become more pertinent following technical innovations in long-read sequencing and super-resolution microscopy. We present a model of centromere organisation for monocentromeres that incorporates additional complexity. We then use this model to reconceptualise diverse centromere forms in other eukaryotes including regional centromeres, holocentromeres and centromeres that lack key proteins including CENP-A. In this way, we hope to move towards a unified understanding of centromeric chromatin.

## Introduction

**Fig. 1:**
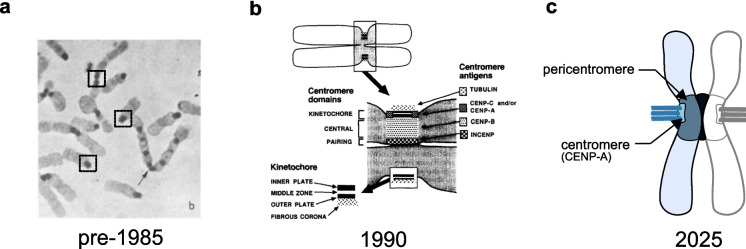
Models of monocentromere organisation over the last fifty years. **a**) Uniform staining of centromeric heterochromatin in mouse cells by C-banding. Boxes highlight some key examples. Image from McKay (1973). **b**) A diversity of chromatin states incorporated into a model of centromere organisation. Image from Pluta et al. (1990). **c**) A ‘canonical’ model of centromeric chromatin defined by the CENP-A-containing region, with the remainder of the primary constriction summarized as the pericentromere

The definition of what constitutes a centromere has gone through a series of expansions and contractions over the past 90 years. Initially proposed in 1936 (Darlington 1936), for the first fifty years most centromeres were defined morphologically as a dense region of chromatin with a single structural specialisation, the kinetochore, at its outer surface (reviewed in Earnshaw 1991). In monocentromeres that are present at a single site per chromosome, including all human chromosomes, this definition includes the entire primary constriction and is premised on two key functions of the centromere being: 1) binding the kinetochore to each sister chromatid and 2) maintaining sister chromatid cohesion in mitosis until anaphase onset (Pluta et al. 1990). Initially, the homogeneity of centromeric chromatin was assumed, based on its uniform heterochromatin staining in monocentromeres (e.g. McKay 1973), and there was no suspicion that centromeres contain sub-structure within the primary constriction (Fig. 1a). However, from the mid 1980’s a series of studies using antibody probes revealed a diversity of chromatin states, including non-overlapping enrichments of human centromere proteins CENP-A, CENP-B and INCENP at the kinetochore domain, central domain and pairing domain respectively (Fig. 1b) (Earnshaw et al. 1986; Cooke et al. 1987; Rattner et al. 1988; Compton et al. 1991). The definition of centromeric chromatin was briefly altered to accommodate this increased complexity and it was envisaged that subsequent high-resolution imaging of novel centromere proteins would capture an increasingly detailed landscape which could inform mechanistic interpretation of centromere functionality (Pluta et al. 1990; Earnshaw and Rattner 1991; Earnshaw 1991). Instead, a reductionist definition of centromeric chromatin based on the localisation of the histone H3 variant CENP-A became dominant, in which the roughly 8% of the primary constriction containing interspersed CENP-A (Marshall et al. 2008) was considered the ‘centromere’ and the rest relegated to a loosely defined term ‘pericentromere’ (Fig. 1c). The problems with this simplification were noted early, including in a review titled ‘When is a centromere not a kinetochore?’ (Earnshaw 1991). However, by concentrating on a smaller region of the primary constriction incredible advances have been made in our understanding of CENP-A chromatin and the inner-kinetochore (Black 2017; McAinsh and Marston 2022). This came at the expense of further characterising chromatin diversity elsewhere in the primary constriction, which has barely advanced since the early 1990s. Thanks to technological advances in super resolution microscopy and long-read DNA sequencing that make high-resolution investigation of chromatin at centromeres widely available (Altemose et al. 2022a, b; Sen Gupta et al. 2023; Sacristan et al. 2024; Kixmoeller et al. 2025), we believe that the time has come to revisit the definition of centromeric chromatin to encompass diverse chromatin states at the primary constriction. In this review we will discuss clear cases of distinct protein localisation within the primary constriction and suggest a general framework for incorporating new protein localisations into a maximalist definition of centromeric chromatin.

## Discovery of centromeric chromatin complexity

**Fig. 2:**
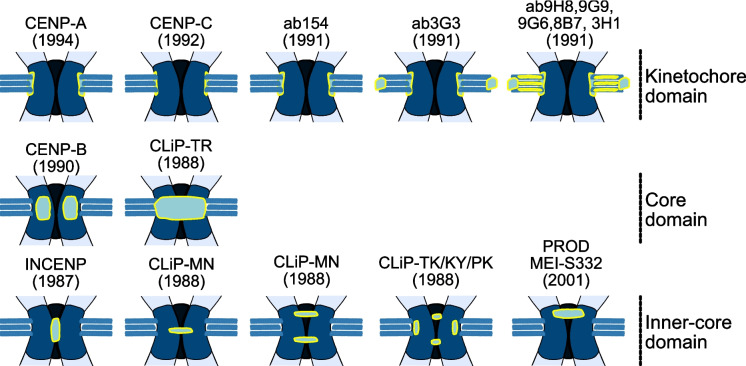
Early antibody staining revealed diverse domain in centromeric structure. Antibody signal illustrated as a cyan distribution surrounded by a yellow outline. Date in brackets identifies the first precise localisation, references below. Ab### refers to a monoclonal antibody for which the specific protein is unknown. In this case centrosomes are also highlighted for some epitopes. CLiP-## refers to patient derived sera, with ## referring to the serum number

A still unexplained observation from the early 1980 s is that patients with systemic scleroderma often express autoantibodies to centromere-specific proteins (known as anticentromere antibodies (ACA)) in their blood (reviewed in Earnshaw 2015). Early characterization of systemic scleroderma antibodies suggested that these antigens all bind the kinetochore forming region with a highly similar localisation (Brenner et al. 1981), which may have contributed to the widespread adoption of a simplified model of centromere organisation (noted in Earnshaw 1991) (Fig. 1c). Subsequent detailed characterisation by immunoelectron microscopy demonstrated that, whereas CENP-A and CENP-C do have a similar localization at the kinetochore (Earnshaw and Rothfield 1985; Saitoh et al. 1992; Sullivan et al. 1994), CENP-B is in-fact not associated with the kinetochore but at least 99% of detected antigens are found buried within the primary constriction in a region the authors called the central domain (Fig. 1b and 2) (Cooke et al. 1990; Marshall et al. 2008). Therefore, results from the first described family of centromere proteins (Earnshaw and Rothfield 1985) demonstrate a diversity of chromatin states defined by anti-centromere antibodies.

Complementing these observations, additional centromere localisations in the region between sister chromatids were described when staining the chromosomes of Indian muntjac with serum from patients with systemic sclerosis (Rattner et al. 1988). These localisations were called ‘Chromatid Linking Proteins’ or CLiPs and most likely detect proteins involved in the maintenance of sister chromatid cohesion (Fig. 2 – labelled CLiP-##).

Soon after these discoveries, a second approach was developed by the Earnshaw and Cleveland labs that fortuitously identified centromere proteins associated in distinct spatial and temporal patterns (Cooke et al. 1987; Compton et al. 1991). These studies injected isolated mitotic scaffolds depleted for nucleosomes into mice, in the hope of generating antibodies that identify novel proteins involved in mitotic chromosomes organisation. The first protein discovered via this approach was INCENP, which binds tightly to mitotic chromosomes independent of nucleosomes and showed a dynamic and novel centromere localisation at the pairing domain between sister chromatids (Cooke et al. 1987) (Fig. 1 and 2). At least four more centromere proteins, each with a molecular weight in excess of 200KDa, were discovered using 11 monoclonal antibodies from a large-scale screening approach (Compton et al. 1991). The minimum number of novel proteins was inferred from the distinct cell-cycle-dependent localisations observed with different antibodies, which showed four combinations of relocalisation between kinetochores and the centrosomes, midbody, spindle poles or diffuse throughout the nuclei in different stages of the cell cycle (Fig. 2 – labelled ab###). Unfortunately, the specific proteins detected by these antibodies remain unknown.

These studies reveal that the complexity of centromeric chromatin was understood from the very earliest characterisation of centromere-specific proteins. However, in the intervening time few studies have attempted to address the specific localisation of other proteins within classical monocentromeres. Isolated examples predating recent advances in super resolution imaging include the constitutive centromere associated network (CCAN) which directly associates with CENP-A nucleosomes (Foltz et al. 2006), the perikinetochoric ring as the site of MKAK association (Parra et al. 2006) and the cohesion domain which sits to one side of the CENP-A chromatin in Drosophila (Blower and Karpen 2001) (Fig. 2, PROD and MEI-S332).

## The shift from technical limitations to technical opportunities

Classically, precise localisation of proteins within cellular structures was performed by immunoelectron microscopy (e.g. Cooke et al. 1990; Marshall et al. 2008), but this technique is technically challenging and was not widely embraced in centromere biology. Instead, lower resolution microscopy approaches based on immunofluorescence and live-cell imaging of fluorescent proteins became routine (Saffery et al. 2000; Chen et al. 2025). For this reason, the definition of centromeric proteins became imprecise or ‘fuzzy’, meaning that proteins which may not co-localise at the molecular scale have been assumed to do so based on their co-localisation by light microscopy.

In subsequent years, approaches that map the precise genomic localisation of a protein of interest have been developed including ChIP-seq and CUT&RUN (Ma and Zhang 2020). These techniques have solved the problem of understanding the localisation of a protein at the molecular-scale within unique sequence DNA, such as those associated with genes and their regulatory domains (Panigrahi and O’Malley 2021). However, centromeric chromatin in most model organisms is built upon highly repetitive DNA that is excluded in most genome builds (Miga 2017; Thakur et al. 2021), and mapping of proteins was therefore not possible within these loci. For this reason, the molecular-scale distribution of most proteins across centromeric loci remains completely unknown.

Recently, microscopy advances are providing new high-resolution insights into the structure of centromeres that include super-resolution microscopy (SRM), expansion microscopy (ExM) and correlative light and electron microscopy (CLEM) (Sen Gupta et al. 2023; Sacristan et al. 2024; Kixmoeller et al. 2025; Zhao et al. 2025). So far, these techniques have been used to investigate CENP-A, the distribution of SMC proteins and ATRX – revealing additional complexity to the canonical models. This includes a previously unrecognised domain located below the CENP-A chromatin that is enriched for cohesin and condensin, and a split profile of cohesin and ATRX enrichment either side of the primary constriction in the inner-centromere. Each of these studies describes the localisation of their protein of modification of interest in their own terms, and there is no unified model for presenting intra-centromere protein localisation and the relation of different protein components to each other. Forthcoming microscopy studies of diverse centromere proteins require a model that integrates older and recent discoveries, in order to illuminate our understanding of the 3D structure and sequential regulation of centromeric chromatin.

Other advances include long read DNA sequencing, which has enabled the completion of reference genome builds that contain highly repetitive centromeric DNA. Telomere-to-telomere builds are now available for key model organisms including human, mouse, chicken and *Arabidopsis thaliana* (Naish et al. 2021; Nurk et al. 2022; Huang et al. 2023; Liu et al. 2024). Long read DNA sequencing allowed for the identification of a dip in DNA methylation that co-localises with CENP-A and has been defined as the Centromere Dip Region (CDR) (Logsdon et al. 2021; Altemose et al. 2022a). Additionally, the existence of accurate maps of centromeric DNA loci allow for the low-confidence mapping of short read ChIP-seq or CUT&RUN data (Logsdon et al. 2021), giving a first approximation as to the distribution of a protein with respect to CENP-A enriched chromatin. For high confidence mapping of chromatin proteins within repetitive centromeric DNA an approach was developed based on introducing non-canonical methylations to DNA in proximity to a protein of interest, followed by long-read sequencing. This method, called DiMeLo-seq, has been used successfully to understand the precise localisation of human CENP-A and H3K9me3 (Altemose et al. 2022b). Expanding these approaches to map diverse proteins has the potential to revolutionise our understanding of the linear distribution and composition of distinct centromeric chromatin domains.

Once the specific molecular-scale co-localisation of proteins within the centromere is confirmed it becomes possible to use recent advances in cryo-electron microscopy (cro-EM) to decipher the precise organisation of centromere-linked components. This approach has been used to solve the structure of a significant part of the inner- and outer-kinetochore structure, including the discovery of novel protein interactions (Yan et al. 2019; Yatskevich et al. 2022; Pesenti et al. 2022; Muir et al. 2023; Polley et al. 2024). However, other regions within the centromere, in particular those associated with CENP-B, remain totally uncharacterised in terms of binding partners and corresponding structural data. A first step towards identifying further centromere-associated structures is to capture the specific co-localisation of known and novel proteins in the cell, and for this an accurate model of local chromatin composition is required.

In summary, we have entered an era in which the complex composition of centromeric chromatin will inevitably be re-discovered and further expanded through the use of new technologies. Therefore, incorporating what we already know into an expanded model of centromeric chromatin will provide an initial benchmark to be used for determining the complete structure and function of these genomic loci.

## Proposed model of centromere organisation for monocentromeres

**Fig. 3:**
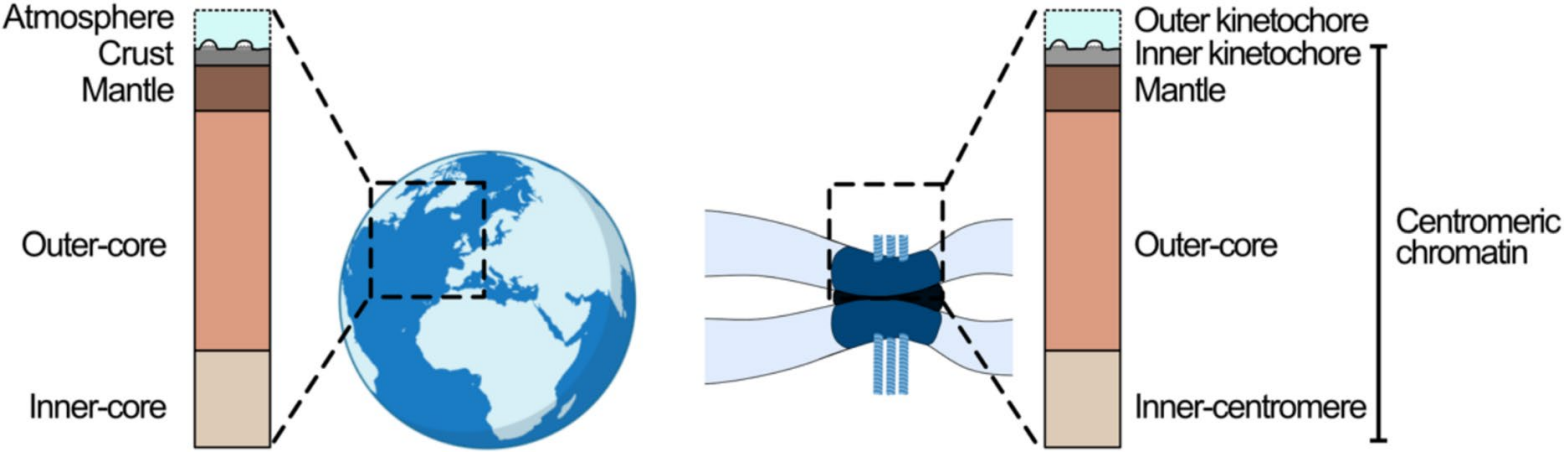
General framework for centromere organisation built on a model of the internal stratification of the Earth

To enable the integration of additional complexity into models of centromeric chromatin we propose to build on previous models published 35 years ago (Pluta et al. 1990; Earnshaw and Rattner 1991) (Fig. 1). Initially, we explicitly focus on better-studied monocentromeres but will later consider how this model influences our interpretations of other centromere forms (Schubert et al. 2020). To provide flexibility for incorporating future discoveries, we take inspiration from the layered internal structure of the Earth in which different layers (e.g. the mantle) can be potentially sub-divided when new discoveries are made in the future (e.g. upper and lower mantle). In our model (Fig. 3, Table 1), we start with the outer-kinetochore as the ‘atmosphere’ – not a part of the chromatin (or internal structure of the Earth) but a necessary component of the whole picture that gives the centromere (and the Earth) its outer appearance. We will then describe four distinct layers moving from the CENP-A enriched inner-kinetochore, through the mantle and the outer-core, towards the inner-centromere which links sister chromatids. Importantly, due to the underappreciated complexity of centromeric chromatin in experimental studies to-date, the size of each domain is unknown with any precision in the linear or 3D chromatin context. We will therefore focus on protein markers which, in combination, delineate distinct chromatin subtypes (Table 1) but refrain from providing indications for sizes or scales. Briefly, these domains can be defined by:

**Table 1:** Marker proteins for each domain of centromeric chromatin with high-resolution experiments referenced

Domain	Proteins	Description of interaction	Reference
**Inner-kinetochore**	CENP-A	H3 variant incorporated into centromere specific nucleosomes	Electron microscopy—Marshall et al. 2008 CLEM—Kixmoeller et al. 2025 Super resolution microscopy—Sacristan et al. 2024, Sen Gupta et al. 2023 DiMeLo-seq—Altemose et al. 2022a Cryo-EM—Pesenti et al. 2022, Yan et al. 2019
	16 subunit CCAN	Key components of the inner-kinetochore that directly bind CENP-A	Cryo-EM—Pesenti et al. 2022, Yan et al. 2019
**Mantle**	Cohesin	minor pool adjacent to the kinetochore (approx 100 nm speration)	Super resolution microscopy—Sacristan et al. 2024, Sen Gupta et al. 2023
	Condensin	co-localised with cohesin pool adjacent to the inner-kinetochore	Super resolution microscopy—Sacristan et al. 2024
	H2AT120ph	approx 100 nm adjacent to inner-kinetohore	Immunofluoresence (low resolution)—Broad et al. 2020
	SgoI	binds H2AT120ph and found kinetochore proximal, mantle domain localisation inferred	Immunofluoresence (low resolution)—Liu et al. 2013
**Central domain**	CENP-B	major domain that does not co-localisae with the inner-kinetochore, matle or inner-centromere	Electron microscopy—Cooke et al. 1990 Super resolution microscopy—Sacristan et al. 2024
**Inner centromere**	Chromosomal passenger complex (INCENP, Borealin, AURKB)	Localised between sister chromatids	Electron microscopy—Earnshaw and Cooke 1991 Cryo-EM—Jeyaprakash et al. 2007
	cohesin	Major localisation between sister chromatids	Super resolution microscopy—Sacristan et al. 2024, Sen Gupta et al. 2023
	AURKB	Major localisation between sister chromatids	Super resolution microscopy—Kouznetsova et al. 2024
	ATRX	split location between sister chromatids	Super resolution microscopy—Zhao et al. 2025
	SgoI	Major localisation between sister chromatids	Immunofluoresence (low resolution)—Liu et al. 2013
	H3T3ph	Major localisation between sister chromatids	Immunofluoresence (low resolution)—Broad et al. 2020

- *Atmosphere*

The outer kinetochore, not part of the chromatin.

- *Inner-Kinetochore*

CENP-A chromatin and directly associated factors that form the inner-kinetochore.

- *Mantle*

Immediately beneath the inner-kinetochore with a distinct enrichment of SMC proteins.

- *Outer-Core*

Largest domain with a poorly characterised composition that includes CENP-B.

- *Inner-Centromere*

Protein components of sister chromatid cohesion establishment and resolution.

### Outer kinetochore (the atmosphere)

The kinetochore mediates the interaction of the mitotic spindle with the chromosomes in prophase to ensure accurate chromosome segregation during mitosis (Musacchio and Desai 2017). Our understanding of kinetochore structure is particularly advanced when compared to the centromeric chromatin. Early studies by electron microscopy identified the tri-laminar structure composed on the outer opaque layer that binds microtubules, the translucent middle layer and the opaque inner layer (reviewed in Earnshaw 1991). Decades of structural work followed which recently culminated in high resolution structures of the outer- kinetochore (Muir et al. 2023; Polley et al. 2024). Further information on kinetochore structure has been covered extensively in recent reviews (Ariyoshi and Fukagawa 2023).

The function of kinetochores is highly conserved and is present in all eukaryotes studied to-date (Ishii and Akiyoshi 2022). When chromosome fusions events such as Robertsonian translocations occur, one of the original centromeric chromatin sites is inactivated and this site does not have a kinetochore (Stimpson et al. 2012). Similarly, neocentromeres on chromosomes that retain the centromeric satellite DNA sequence only assemble kinetochores on the neocentromere and not at the original repetitive DNA site (DeBose-Scarlett and Sullivan 2021). Therefore, the most-conserved feature of a functional centromere is that it has an associated kinetochore, which acts as a microtubule binding site. However, the outer-kinetochore is not a part of the centromere as it contains no DNA or chromatin (Cooke et al. 1993).

### Inner-kinetochore domain (the crust)

The inner-kinetochore domain is composed of chromatin that is associated with the inner-kinetochore plate, and is typically enriched for CENP-A. This chromatin does not extend into other layers of the kinetochore or into other parts of the primary constriction (Cooke et al. 1993). Similarly to the crust of the Earth, the inner-kinetochore is a minor part of the overall structure of the primary constriction. The precise localisation of CENP-A within human chromosomes demonstrated that only 6–8% of the total volume of the primary constriction is enriched for CENP-A (Marshall et al. 2008). Importantly, this CENP-A enriched chromatin does not co-localise with other well-characterised components of the centromere including CENP-B or the inner-centromere. Instead, the key interacting proteins of CENP-A in mammals form the CCAN, which is also restricted to the inner-kinetochore and includes CENP-C (Foltz et al. 2006).

The CCAN is a 16 subunit protein complex that is found associated with CENP-A chromatin throughout the cell cycle (Pesenti et al. 2022). Recent work has identified a pool of cohesin that directly interacts with the CCAN through a direct interactions with CENP-U, a component of the CENP-OPQUR sub-complex (Yan et al. 2024). The inner-kinetochore associated cohesin forms a minor portion of the overall pool of these proteins on mitotic chromosomes which can be identified biochemically but has not been observed by super-resolution microscopy (Sen Gupta et al. 2023).

The precise localisation of the inner-kinetochore was first established in the genome of *S. cerevisiae*, which has a highly unusual ‘point centromere’ encoded by a specific 125 bp DNA sequence (Carbon and Clarke 1984; Meluh et al. 1998). We refer readers to literature that cover this exceptional monocentromere example (Kobayashi et al. 2015; Friedman and Freitag 2017; Talbert and Henikoff 2020). To determine the localisation of CENP-A in other organisms with kilobase to megabase sized monocentromeres, naturally occurring unique sequence centromeres have been utilised including the Z chromosome in chicken (Sacristan et al. 2024), chromosome 11 in horse (Cappelletti et al. 2023), 13 of 23 centromeres in a species of Zebra (Cappelletti et al. 2022) and naturally occurring neocentromeres in human and other organisms (DeBose-Scarlett and Sullivan 2021). Furthermore, artificial models for exploring neocentromeres have been generated including systems in which the endogenous centromere was cleaved from the chromosome (Murillo-Pineda et al. 2021) and human-hamster hybrid cell lines that contain single human chromosomes with neocentromeres (Naughton et al. 2022). These studies identify CENP-A domains of around 25–300 Kbp, which can locally reposition within around 500 Kbp and which are co-localised with a dip in the heterochromatin marks H3K9me3 and DNA methylation. Earlier microscopy studies indicate that the linear genome is arranged in discrete patches enriched for CENP-A interspersed with patches containing only regular H3 nucleosomes (Sullivan and Karpen 2004). It is believed that these adjacent patches fold in three-dimensions to expose CENP-A nucleosomes to the kinetochore, and a long-standing challenge is how the linear arrangement of centromeric DNA corresponds to the 3D structure of the centromeric chromatin and how it changes through the cell cycle (Vargiu et al. 2017). It is now possible to address this question directly by using recent advances in genomics and microscopy, but conceptual advances are necessary to interpret potentially complex experimental outputs (see future directions).

To date, studies utilising advanced genomic and molecular techniques in centromeric chromatin have focussed on the inner-kinetochore, which is the only domain that has been investigated using long-read sequencing, super resolution microscopy, CLEM and cryo-EM. DiMeLo-seq confirms properties of CENP-A identified in unique-sequence centromeres and, together with other forms of long read sequencing, identified and named the CDR and showed that H3K9me3 is mutually exclusive with CENP-A containing chromatin (Naish et al. 2021; Altemose et al. 2022b; Liu et al. 2024). Detailed analysis of long read Fiber-seq data identified an unusual nucleosome distribution within CENP-A chromatin called dichromatin, in which accessible chromatin patches are interspersed with protected chromatin with the size of a dinucleosome (Dubocanin et al. 2025). Dichromatin was not observed outside of the CENP-A domain within the broader centromere, or at other sites in the genome, suggesting the existence of a specific kinetochore-associated chromatin organisation. In addition, SRM, ExM and CLEM have all identified that single centromere positions observed by light microscopy are in fact composed of bipartite CENP-A domains tethered together to form a single inner-kinetochore subunit (Sacristan et al. 2024; Kixmoeller et al. 2025). Finally, cryo-EM has identified the complete structure of the CCAN bound to a CENP-A nucleosome in human and yeast at almost atomic resolution (Yan et al. 2019; Yatskevich et al. 2022; Pesenti et al. 2022). Together, these first studies integrating recent high-resolution approaches have demonstrated that novel properties of centromere organisation can be discovered, even for the best studied centromeric chromatin domain.

### Mantle domain

Adjacent to or buried underneath the inner-kinetochore is a domain identified in many independent studies and which is associated with a distinct pool of proteins that are more typically associated with sister chromatid cohesion in the inner-centromere, including cohesin, condensin, H2AT120ph and SgoI (Parra et al. 2006; Liu et al. 2013; Broad et al. 2020; Abad et al. 2022; Sen Gupta et al. 2023; Sacristan et al. 2024). The first hint of this domain came from the localisation of CLiP proteins TK, KY and PK (Fig. 2) (Rattner et al. 1988). This structure cannot be accurately resolved from the inner-kinetochore using standard light microscopy approaches, but super resolution and expansion microscopy have both demonstrated a distinct domain immediately adjacent to but beneath the inner-kinetochore (Sen Gupta et al. 2023; Sacristan et al. 2024). Precise measurement of this domain indicates that it sits approximately 100 nm from the inner-kinetochore domain (Sen Gupta et al. 2023). We call this region the ‘mantle domain’ and it remains unclear if this is one structure or a series of distinct layers in the chromatin (which could then be named inner- and outer- mantle, or further subdivided) (Sacristan et al. 2024; Andrade Ruiz et al. 2024). We hypothesise that the mantle domain has important regulatory activity in centromere structure and function.

### Outer-core domain

The outer-core domain represents most of the primary constriction in monocentromeres and is best characterised in human and Indian muntjac (Rattner et al. 1988; Pluta et al. 1990). Physically it occupies the space between the inner-kinetochore/mantle domain and the inner-centromere. The function of the outer-core is unclear and only a single non-essential protein has been identified in mammalian centromeres, the key autoantigen CENP-B (Cooke et al. 1990). The outer core-domain may provide structural integrity to the centromere during chromosome segregation or may help position the kinetochore-domain chromatin by providing an optimal environment for CENP-A loading and the establishment of the kinetochore (Okada et al. 2007; Chardon et al. 2022). It may also act as a buffer region between the kinetochore- or mantle-domain and the inner-core domain.

Both CENP-B protein and the CENP-B box DNA motif are highly conserved, but CENP-B depletion has minimal effect on centromere structure or function in chromosome segregation (Gamba and Fachinetti 2020). CENP-B is composed of a centromere-specific DNA binding domain and a dimerization domain, and together these properties can generate loops in centromeric satellite DNA (Chardon et al. 2022). One possibility is that there are additional uncharacterised proteins that redundantly structure looped domains in outer-core chromatin. Potential candidates include highly abundant centromere-targeting HMG proteins (Disney et al. 1989) or linker histone variants (Saha et al. 2025), which are able to cross-link and/or promote chromatin interactions. Additionally, condensin complexes, which extrude loops and structure chromosomes (Cutts and Vannini 2020), have already been established to organise the relative position of the inner-kinetochore and inner-centromere (Samoshkin et al. 2009). Independent lines of evidence suggest that CENP-B is stabilised in its interaction with the centromere during mitotic progression (Hemmerich et al. 2008; Chen et al. 2025), indicative of a functionally conserved timing for loop generation via distinct pathways. Characterising these pathways, or which other proteins are abundant and function in the outer-core, remains to be investigated.

In addition to a direct role on structure, CENP-B also performs an epigenetic rescue of centromeres. In centromeres that have lost CENP-A, through experimental perturbation or in long-term quiescent cells, CENP-B is necessary for the re-incorporation of newly synthesised CENP-A (Hoffmann et al. 2020). Similarly, human artificial chromosomes require CENP-B protein and CENP-B box DNA sequences in order to establish de novo centromeres (Ohzeki et al. 2002). In newly established human chromosome fusions with two centromeres, CENP-B is required to form a functional centromere at one alpha-satellite site and heterochromatinise the other non-functional site (Okada et al. 2007). How CENP-B promotes CENP-A loading is unclear, particularly as these proteins do not normally co-localise at the molecular-scale in cells (Cooke et al. 1990; Marshall et al. 2008). Understanding the mechanistic basis linking the outer-core domain, CENP-B and epigenetic rescue is a particularly interesting direction for future studies.

The outer-core domain also represents at least part of the classical pericentromeric heterochromatin. Chromatin fibre mapping experiments have shown that the kinetochore adjacent chromatin is enriched for heterochromatic marks (Sullivan and Karpen 2004; Altemose et al. 2022b). However, CENP-B has a strong preference for unmethylated CpGs within its DNA binding sequence (CENP-B box), but unmethylated DNA is not typically present within constitutive heterochromatin (Tanaka et al. 2005). It is therefore conceivable that within the outer-core there are distinct domains of different chromatin composition that promote CENP-B association or heterochromatin formation, but which have not been observed by low-resolution approaches used to investigate centromeric chromatin thus far. Alternatively, some of the CENP-B chromatin may occur in patches of H3 chromatin within the CDR, which then loop out from the adjacent CENP-A patches to form the outer-core. To address the relationship between the linear and 3D chromatin organisation of the outer-core, it is now necessary to fully characterise the localisation of CENP-B using long-read chromatin mapping and super-resolution imaging approaches.

### Inner-centromere domain (Inner-core)

The inner-centromere domain is the site that links sister chromatids. It contains a diversity of described localisations whose relative overlap and distribution remain completely uncharacterised in any form of centromere organisation.

The first protein of a distinct chromatin sub-type between sister chromatids was identified with the discovery of INCENP in human chromosomes (Cooke et al. 1987; Earnshaw and Cooke 1991), which was later shown to be a key component of the chromosome passenger complex (Carmena et al. 2012). Subsequently, other components of the chromosomal passenger complex, the cohesion domain, enriched condensin localisation, the localisation of the CLiPs antigens and others have been described to occur between sister chromatids, but it is not clear whether these represent a single domain or a whole series of distinct domains (Earnshaw et al. 1986; Cooke et al. 1987; Rattner et al. 1988; Compton et al. 1991; Kouznetsova et al. 2024). For example, cohesin subunits and CLiPs have been shown to localise either side of the primary constriction, but the cohesion domain described in *Drosophila* is present on just one side of the primary constriction (Rattner et al. 1988; Blower and Karpen 2001). Recent super resolution microscopy demonstrated that the chromatin remodeller ATRX binds the inner-core either side of the primary constriction and the authors propose that the bipartite structure observed at the inner-kinetochore is present throughout the centromere, consistent with recent models (Zhao et al. 2025). Cohesin has been shown by super resolution microscopy to bind inner-centromeres with three different distributions within the same chromosome spread, categorised as ‘split’, ‘uniform’ or ‘central’, and the authors propose that these classes are determined by the amount of centromeric DNA incorporated into the inner-centromere (Sen Gupta et al. 2023). In general, the diversity of protein localisation observed in the inner-kinetochore remains poorly characterised and we have no idea if these are structurally distinct domains, organism specific variation in otherwise conserved structures, cell-type specific properties or chromosomes specific observations. Despite these uncertainties, the functional contribution of proteins in this region is well established at molecular detail, including structures of the core components of the chromosome passenger complex (Jeyaprakash et al. 2007). Furthermore, this domain contains the only centromere-associated proteins with clear orthologs in Trypanosomes, demonstrating conservation across all described eukaryotic phyla (Ballmer and Akiyoshi 2024; Ballmer et al. 2024a, b). Defining spatial and temporal maps at the molecular scale for the inner-core domain protein will enable the development of better functional models of chromosome segregation.

Additionally, we note a commonality between the proteins involved in the mantle domain and the inner-centromere domain that could indicate functional overlap, or even functional redundancy, between these two domains (Table 1). Strikingly, in a *Xenopus* extract system the major pool of centromere cohesin is not at the inner-core, but is instead enriched at the inner-kinetochore, probably in the mantle domain but conceivably in the inner-kinetochore itself (Haase et al. 2025). Understanding why proteins that can stabilise cohesion between chromosomal positions, provide structural rigidity to centromeric chromatin, sense tension generated by the mitotic spindle and signal the spindle assembly checkpoint are localised in at least two structurally distinct regions of monocentromeres will inform additional functional interpretation.

## Eukaryote-wide centromere diversity in the context of complex chromatin

**Fig. 4:**
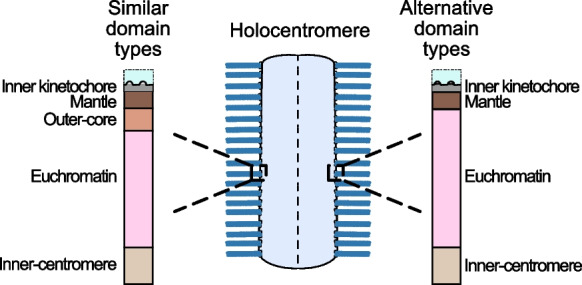
Models of holocentromere organisation indicating potential domain distributions. Detailed experimental comparisons of the protein composition of linear and 3D centromeric chromatin across diverse species is required to substantiate and expand these models

Most studies of centromeres focus on human-like monocentromeres, but other forms of centromere organisation were defined as early as 1935 when the first holocentric insect chromosomes were described (Schrader 1935). Considering centromeres are a universal trait of eukaryotic chromosome organisation, studying diverse centromere forms will help to better understand the necessary and sufficient components that drive kinetochore assembly, sister chromatid cohesion and accurate chromosome segregation in mitosis. For example, in the case of the holocentromeres in *Chionographis japonica*, centromeric units marked by CENP-A/CENH3 are distributed along the whole chromosomes, which cluster themselves on the pole-ward surface of the chromosomes during mitotic metaphase, thereby enabling the microtubule attachment (Kuo et al. 2023). The euchromatin is organised beneath the CENP-A/CENH3 domain, followed by a domain mediating sister chromatid cohesion. Whether a multi- layered centromeric chromatin structure exits between the inner-kinetochore and euchromatin domains is currently unclear (Fig. 4). In part, this reflects our ignorance of the importance of different chromatin sub-types within all forms of centromere organisation. As a first step, we here attempt to incorporate discoveries across diverse species with different types of centromere into the complex chromatin model outlined above (Table 2).

**Table 2:** Published evidence supporting chromatin localisations in diverse centromere forms of centromere organisation

Domain	Proteins	Spcies and centromere diversity	Reference
**Inner-kinetochore domain**	CENH3	monocentric species of plants e.g. *Arabidopsis thaliana*	Naish et al. 2021
		holocentric species of plants e.g. *Rhynchospora pubera, Chionographis japonica*	Hoffstatter et al. 2022; Kuo et al. 2023
		metapolycentric species of plants e.g. *Pisum sativum*	Neumann et al. 2016
**Mantle domain**	H2AT120ph cohesion mark	monocentric species of plants e.g. *Hordeum vulgare*	Schubert et al. 2020
		metapolycentric species of plants e.g. *Pisum sativum*	
		holocentric species of plants e.g. *Rhynchospora pubera*	
**Central domain**	CENP-B	monocentric species of yeast, *Schizosaccharomyces pombe*	Baum and Clarke 2000; Nakagawa et al. 2002
		holocentric species of insects, *Spodoptera frugiperda*	d’Alençon et al. 2011
		monocentric species of plants, beans, carrots, onions and potatoes	Barbosa-Cisneros and Herrera-Esparza 2002
**Mantle/inner core domain**	INCENP	monocentric species of plant, *Arabidopsis thaliana*	Komaki et al. 2020
	H3T3ph cohesion mark	monocentric species of plant, *Zea mays L*	Liu et al. 2017
		holocentric species of plant, *Chionographis japonica*	Kuo et al. 2023
	H3S28ph cohesion mark	monocentric species of plants e.g. *Hordeum vulgare*	Houben et al. 2007
		holocentric species of plants e.g. *Chionographis japonica*	Kuo et al. 2023

### Inner-kinetochore

Following the initial discovery of CENP-A (Earnshaw & Rothfield 1985), ACA was used to detect kinetochores in a very distant plant species, *Scadoxus multiflorus subsp. Katharinae* (previously as *Haemanthus katherinae Bak*), highlighting the remarkable conservation of centromere components (Mole-Bajer et al. 1990). ACA likely stained homologs of CENP-A and CENP-C in *S. multiflorus*, providing compelling evidence for the conservation of both outer- and inner-kinetochore domains. Importantly for understanding the primary literature in plants (and some insects) an alternative nomenclature is in place in which CENP-A is referred to as CENH3 (Talbert et al. 2002, 2012; Earnshaw et al. 2013). CENP-A/CENH3 homologs were soon found to be conserved in many monocentromeres, holocentromeres, metapolycentromeres and neocentromeres (Neumann et al. 2016; Hofstatter et al. 2022; Naish and Henderson 2024). This deep conservation is consistent with a model in which CENP-A/CENH3 defines the position of centromeric chromatin across eukaryotes.

In contrast to this view, there are now many examples of organisms that do not encode a CENP-A homolog. CENP-A/CENH3 is absent in the insect orders, Lepidoptera, Hemiptera, Phthiraptera, Dermaptera and Odonata (Drinnenberg et al. 2014), which underwent at least four independent transition from mono- to holocentricity (Melters et al. 2012). Further experimental validation using Lepidoptera as a model showed that centromere function is conserved in a CENH3-CENP-C independent but CENP-T dependent manner (Cortes-Silva et al. 2020). CENH3 is duplicated in the holocentric plant *Cuscuta* (*C. epithymum* and *C. europea*) but, it does not co-localize with the microtubule attaching sites (Oliveira et al. 2019) or co-localise with CCAN and KMN components (Neumann et al. 2023). In kinetoplastids, which are a very distant eukaryotic ancestor that includes Trypanosomes, there are also no homologs of CENP-A (Akiyoshi and Gull 2014). On this basis, diverse centromeres forms indicate that the inner-kinetochore domains should be defined solely by the functional interaction with microtubules, irrespective of the proteins mediating this interaction, aligning with Darlington’s (1936) original description of centromeres as 'spindle fibre attachment chromosomes'.

### Mantle domain

In human monocentromeres, the mantle domain is proposed to consist of cohesins, condensins, H2AT120ph, and Sgo1, with a potential role in organizing CENP-A on the poleward surface during metaphase. Similarly, the presence of SMC proteins around centromeric nucleosomes is well established in plants. In holocentric species such as *Rhynchospora pubera*, and *Luzula elegans*, centromeric units from a single chromosome display a scattered distribution during interphase. During metaphase, these units cluster together and form a line-like distribution along the poleward surface of the chromosome (Nagaki et al. 2005; Marques et al. 2015). Polymer modeling has suggested that interactions between centromeric nucleosomes and SMC proteins are essential for this higher-order organization (Câmara et al. 2021), thereby supporting the presence of SMC proteins in proximity to CENH3/CENP-A proteins. Consistently, H2AT120ph exhibits a line-like distribution on the poleward surface, distinct from the euchromatin domain, in the holocentric species *L. elegans* (Demidov et al. 2014). H2AT120ph also shows centromeric localization in 20 analyzed plant species—including mono-, meta-, poly-, and holocentric species—and serves as a universal marker for plant centromeres (Demidov et al. 2014; Schubert et al. 2020). High-resolution structured illumination microscopy in the monocentric plant species *Hordeum vulgare* revealed that CENH3/CENP-A and H2AT120ph occupy only a portion of the primary constriction, and importantly, they are located in distinct domains (Demidov et al. 2014). This provides further support for the proposed multi-domain model and suggests its potential conservation across species and diverse centromere types.

### Outer-Core

Functional homologs/analogs of CENP-B have been reported across various eukaryotic lineages, including the yeast *Schizosaccharomyces pombe* (Baum and Clarke 2000; Nakagawa et al. 2002), several insects like *Spodoptera frugiperda* (d’Alençon et al. 2011), and plants like *Phaseolus vulgaris* (Barbosa-Cisneros and Herrera-Esparza 2002) as a result of convergent evolution. In *S. pombe*, CENP-B analogs bind centromeric repeats and play a role in the formation of centromeric heterochromatin (Baum and Clarke 2000; Nakagawa et al. 2002). In the holocentric insect species, *Spodoptera frugiperda*, CENP-B binding sequences are distributed across the entire genome, consistent with their holocentric nature (d’Alençon et al. 2011). However, any direct centromeric role has not been reported. Considering that CENP-B is the only DNA-specific centromeric protein, its presence across diverse eukaryotes and centromere types hints at the possibility of a conserved feature of the outer-core domain.

### Inner-Core Domain

The components of the inner-core domain are not well characterized outside of mammals and yeast. However, scattered evidence suggests conservation of key components across eukaryotes. Homologs of INCENP and AURKB have been reported in *Arabidopsis thaliana* and trypanosomes (Komaki et al. 2020; Ballmer & Akiyoshi 2024). Notably, Borealin homologs are present across plant lineages, from algae to angiosperms (Komaki et al. 2020). In humans, CPC localization to the inner centromere of metaphase chromosomes is mediated by the histone mark H3T3ph (Broad et al. 2020). Similarly, H3T3ph also shows inner-core localization in plants. In maize, H3T3ph localizes to centromeres during metaphase, is absent from inactive centromeres of dicentric chromosomes, and is proposed to stabilize pericentromeric cohesion (Liu et al. 2017). In the metaphase chromosomes of the holocentric species *Chionographis japonica*, H3T3ph localizes to the inner core of the chromosomes at the sites of sister chromatid attachment. Consistent with its holocentric nature, the distribution spans the entire chromosome, suggesting functional conservation of sister chromatid cohesion in diverse centromere types (Kuo et al. 2023). Importantly, in holocentric species, euchromatin is organized between the inner-kinetochore/mantle domain and the inner-core domain (Fig. 4). In this context, the inner core appears to function independently of the centromeric structure. Only comprehensive studies across diverse centromere types will be able to resolve centromere architecture and clarify the relationships between these domains.

## Conclusions and future directions

It has long been understood that the centromere is a complex chromatin environment composed of distinct protein localisations, but this diversity is typically downplayed in favour of a reductionist model composed of the kinetochore-associated ‘centromere’ and heterochromatin-associated ‘pericentromere’. In this review, we build on the foundations established by early discussions of centromere autoantigens and propose a model with four chromatin domains (Fig. 3). Our model aims to capture all of the specific localisations that have been identified in monocentromeres thus-far and leaves room for the incorporation of greater detail as it is discovered by subdividing these domains. This reframing is appropriate for considering whether similar domains dominate in diverse forms of centromere organisation, including monocentromeres, metapolycentromeres and holocentromeres (Table 2 and Fig. 4). By incorporating complexity, we build on an earlier definition of centromeres that includes all established domains with centromere-associated function in kinetochore establishment, sister chromatid cohesion and chromatin-associated mitotic checkpoints (Fig. 1 and 3) instead of just the CENP-A region. In this way, we reiterate that a centromere is far more than the inner-kinetochore. The necessity of such a model may be most obvious by highlighting unresolved questions of centromere structure and function:

### How can we reconcile linear and higher-order chromatin organisation in the centromere?

**Fig. 5:**
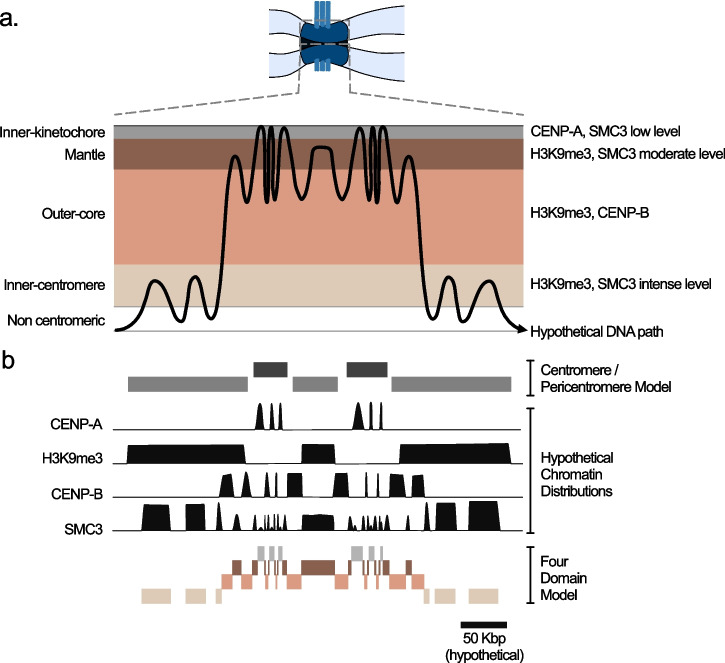
Conceptual model of the relationship between linear and higher-order chromatin organisation in the four-state model. **a**) Hypothetical path of DNA through the distinct chromatin domains resulting from the 3D folding of the chromatin fibre in the centromere. **b**) Corresponding hypothetical chromatin protein mapping along the linear DNA fibre. The ‘hypothetical chromatin distributions’ are example patterns that might be achieved by long-read sequencing approaches in repetitive DNA, demonstrating that key proteins give patterns that are hard to interpret unless considered within the 3D context. The classical ‘centromere/pericentromere’ and four domain model (colours match Fig. 5a) are highlighted

Established models of centromeric chromatin organisation propose that the chromatin/DNA fibre loops into a complex higher-order conformation (Vargiu et al. 2017). This means that linearly adjacent DNA regions are assembled into distinct chromatin configurations, which we represent as the hypothetical path of the DNA through a conceptual model of our four-domain centromeric chromatin classification (Fig. 5a). Current molecular-scale mapping of chromatin proteins within repetitive DNA using DiMeLo-seq is limited to CENP-A and H3K9me3 (Altemose et al. 2022b) and these proteins fit neatly into the ‘centromere’/pericentromere dichotomy by spanning reasonably contiguous blocks with mutually exclusive enrichment (Fig. 5b represents a hypothetical distribution of these proteins). However, domains containing CENP-B in the outer-core and three distributions of SMC3/cohesin (intense at inner-centromere, moderate at mantle, low at the inner-kinetochore), established through microscopy studies (Cooke et al. 1990; Sen Gupta et al. 2023; Sacristan et al. 2024; Yan et al. 2024), give a complex linear chromatin pattern that is extremely difficult to interpret without higher-order context (Fig. 5b). CENP-B appears to approximately co-localise with CENP-A but with a slightly broader organisation. However, by overlaying the four-domain information and focusing on the precise positions of the peaks we can in fact see that CENP-B never co-localises with CENP-A, but instead forms distinct domains which are always buffered by the mantle domain. SMC3/cohesin initially appears to localise throughout the centromere region, with a higher intensity in the outer positions. Again, overlaying the four-domain model gives a fundamentally different interpretation. In this case, SMC3/cohesin is excluded from the outer-core which represents a major part of the centromere in 3D that is dispersed through the linear chromatin fibre. In each of the three remaining chromatin domains there are domain-specific intensities of SMC3/cohesin enrichment, but again these might be obscured by only considering the linear distribution of chromatin. Therefore, by focusing on marks that are distributed in the broadest possible manner in repressive chromatin, like H3K9me3 or DNA methylation, a diversity of other chromatin states are masked.

We reiterate that this model (Fig. 5) is conceptual and propose it to highlight how linear chromatin maps can obscure structurally and functionally important information. Experimental data, generated through long-read sequencing approaches, will allow this conceptual model of higher-order chromatin organisation to transition to a data-based model that can be corroborated by identifying the precise 3D localisation of linearly co-localising proteins through super resolution microscopy. Expanding this analysis to a broader diversity of known centromere proteins, and novel centromere proteins as they are discovered, will clarify the precise composition, number and boundary elements of distinct compartments within centromeric chromatin. Alongside the identification of key principles for the linear and 3D chromatin organisation of monocentromeres, similar characterisation must proceed for diverse forms of centromere organisation in order to establish general principles of centromere structure and function.

### What boundary elements distinguish chromatin domains within the centromere?

Within the centromere there are transitions between distinct chromatin sub-types, but the nature of these boundaries is incompletely defined and the factors that regulate boundaries remain uncharacterised. In our four-domain model we recognise multiple boundaries, adding nuance and depth that helps clarify the nature of the boundaries under consideration by long-read sequencing approaches.

### Does all pericentromeric heterochromatin contribute to the functional centromere?

Human inner-kinetochore chromatin typically forms on alpha-satellite DNA, but there are enormous blocks of other adjacent pericentromeric satellite DNA repeats on some chromosomes (HSATII, SST1, etc.) which do not form the inner-kinetochore but do form heterochromatin (Altemose et al. 2022a). In the two-state model these repeats are contiguous with the heterochromatin in the alpha satellite and presumably constitute a part of the pericentromeric heterochromatin, but are these repeats an important part of centromere structure and function? If we consider centromere function to be restricted to the four domains discussed in our model, linked to kinetochore formation and centromere cohesion in mitosis, it may be that adjacent satellite repeats and/or some of the alpha-satellite are not an important component of centromere function, and that they instead accumulate for unrelated evolutionary reasons such as suppression of meiotic recombination (Vincenten et al. 2015). Accurately mapping key proteins from each of the four-domains in our model through repetitive heterochromatin will allow the position and boundary of the ‘functional’ centromere to be addressed, and differentiated from adjacent heterochromatin.

### What is the composition and role of the outer-core domain?

A major component of the centromere is the outer-core, which in most mammals is specifically bound by CENP-B. To date, there have been no studies specifically investigating this major constituent of centromeric chromatin. Further characterisation of outer-core proteins can inform the function of this domain, including determining functional redundancy, and potentially identify the mechanistic basis through which CENP-B regulates de novo CENP-A loading.

### Are there distinct protein domains within the inner-centromere and how do these change through mitosis?

Low resolution microscopy of individual proteins and antigens within the inner-centromere indicates the existence of distinct structural features (Fig. 2). Future work should aim to identify and stratify inner-centromere proteins.

### What is the relationship between the chromatin structure of diverse centromere forms?

The ‘centromere’/pericentromere dichotomy, with megabase sized blocks of repressive heterochromatin, is most obvious in monocentromeres. Current perspectives attempt to squeeze diverse centromere organisation into this model. Instead we believe that a more complex definition of centromeric chromatin encompassing different domains, with distinct protein localisation and functionality, will identify commonalities across centromere subtypes that unify our understanding of centromere structure and function. This will necessitate super resolution microscopy and chromatin protein mapping by DNA sequencing for matched chromatin proteins and modifications across diverse species.

## Data Availability

No datasets were generated or analysed during the current study.
